# High-Efficiency Transduction of Primary Human Hematopoietic Stem Cells and Erythroid Lineage-Restricted Expression by Optimized AAV6 Serotype Vectors *In Vitro* and in a Murine Xenograft Model *In Vivo*

**DOI:** 10.1371/journal.pone.0058757

**Published:** 2013-03-14

**Authors:** Liujiang Song, Xiaomiao Li, Giridhara R. Jayandharan, Yuan Wang, George V. Aslanidi, Chen Ling, Li Zhong, Guangping Gao, Mervin C. Yoder, Changquan Ling, Mengqun Tan, Arun Srivastava

**Affiliations:** 1 Experimental Hematology Laboratory, Department of Physiology, School of Basic Medical Sciences, Central South University, Changsha, China; 2 Shenzhen Institute of Xiangya Biomedicine, Shenzhen, China; 3 Division of Cellular and Molecular Therapy, Department of Pediatrics, University of Florida College of Medicine, Gainesville, Florida, United States of America; 4 Powell Gene Therapy Center, University of Florida College of Medicine, Gainesville, Florida, United States of America; 5 Genetics Institute, University of Florida College of Medicine, Gainesville, Florida, United States of America; 6 Department of Haematology, Christian Medical College, Vellore, Tamil Nadu, India; 7 Center for Stem Cell Research, Christian Medical College, Vellore, Tamil Nadu, India; 8 Department of Traditional Chinese Medicine, Changhai Hospital, Second Military Medical University, Shanghai, China; 9 Shanghai University of Traditional Chinese Medicine, Shanghai, China; 10 Gene Therapy Center, University of Massachusetts Medical School, Worcester, Massachusetts, United States of America; 11 Department of Pediatrics, University of Massachusetts Medical School, Worcester, Massachusetts, United States of America; 12 Department of Microbiology & Physiology Systems, University of Massachusetts Medical School, Worcester, Massachusetts, United States of America; 13 Herman B Well Center for Pediatric Research, Indiana University School of Medicine, Indianapolis, Indiana, United States of America; 14 Department of Pediatrics, Indiana University School of Medicine, Indianapolis, Indiana, United States of America; 15 Department of Molecular Genetics and Microbiology, University of Florida College of Medicine, Gainesville, Florida, United States of America; 16 Shands Cancer Center, University of Florida College of Medicine, Gainesville, Florida, United States of America; Southern Illinois University School of Medicine, United States of America

## Abstract

We have observed that of the 10 AAV serotypes, AAV6 is the most efficient in transducing primary human hematopoietic stem cells (HSCs), and that the transduction efficiency can be further increased by specifically mutating single surface-exposed tyrosine (Y) residues on AAV6 capsids. In the present studies, we combined the two mutations to generate a tyrosine double-mutant (Y705+731F) AAV6 vector, with which >70% of CD34^+^ cells could be transduced. With the long-term objective of developing recombinant AAV vectors for the potential gene therapy of human hemoglobinopathies, we generated the wild-type (WT) and tyrosine-mutant AAV6 vectors containing the following erythroid cell-specific promoters: β-globin promoter (βp) with the upstream hyper-sensitive site 2 (HS2) enhancer from the β-globin locus control region (HS2-βbp), and the human parvovirus B19 promoter at map unit 6 (B19p6). Transgene expression from the B19p6 was significantly higher than that from the HS2-βp, and increased up to 30-fold and up to 20-fold, respectively, following erythropoietin (Epo)-induced differentiation of CD34^+^ cells *in vitro*. Transgene expression from the B19p6 or the HS2-βp was also evaluated in an immuno-deficient xenograft mouse model *in vivo*. Whereas low levels of expression were detected from the B19p6 in the WT AAV6 capsid, and that from the HS2-βp in the Y705+731F AAV6 capsid, transgene expression from the B19p6 promoter in the Y705+731F AAV6 capsid was significantly higher than that from the HS2-βp, and was detectable up to 12 weeks post-transplantation in primary recipients, and up to 6 additional weeks in secondary transplanted animals. These data demonstrate the feasibility of the use of the novel Y705+731F AAV6-B19p6 vectors for high-efficiency transduction of HSCs as well as expression of the b-globin gene in erythroid progenitor cells for the potential gene therapy of human hemoglobinopathies such as β-thalassemia and sickle cell disease.

## Introduction

Hemoglobinopathies, such as β-thalassemia and sickle cell disease, are by far the most common monogenic diseases that afflict humans worldwide, with an incidence rate of 1∶600. These diseases are also the ideal targets for the potential gene therapy, if high-efficiency transduction of HSCs, and erythroid lineage-restricted expression of the human β-globin gene can be achieved. Indeed, recombinant lentiviral vectors were recently shown to mediate β-globin gene transfer and transgene expression in an adult patient with severe β-thalassemia, which led to transfusion-independence [1]. Unfortunately, however, the observed therapeutic benefit was also compromised by transcriptional activation of a cellular proto-oncogene, HMGA2 and clonal expansion of myeloid cells. Thus, it is important to develop alternatives to lentiviral vectors. Recombinant vectors based on a non-pathogenic human virus, the adeno-associated virus 2 (AAV2) have been developed and shown to be safe and effective in a number of recent clinical trials [2], [3]. Others and we have generated recombinant AAV2-globin vectors [4]–[8], but the transduction efficiency of these vectors in primary human HSCs has not been evaluated.

More recently, up to 12 additional AAV serotype vectors have become available [9]–[11], and we and others have documented that AAV6 is the most efficient serotype among AAV1 through AAV12 in transducing primary human CD34^+^ cells [12]–[14]. We and others have also documented that site-directed mutagenesis of specific surface-exposed tyrosine (Y) residues on AAV serotype capsids leads to higher transduction efficiency both *in vitro* and in *vivo* in various cell types [15]–[20], and that Y705 and Y731 single-mutants are capable of transducing primary human CD34^+^ cells more efficiently than their WT counterpart [14].

In the present studies, we combined both these mutations to generate a tyrosine double-mutant (Y705+731F) self-complementary (sc) AAV6 vector to evaluate whether the transduction efficiency in primary human CD34^+^ cells could be further augmented. In addition, we also compared the transcriptional potential of the following two erythroid cell-specific promoters: (i) HS2-βbp [21], [22], and (ii) B19p6 [23]–[28], both *in vitro* and in a murine xenograft model *in vivo*.

We report here that the transduction efficiency of the Y705+731F double-mutant scAAV6 vectors is significantly higher than that of the single-mutant or the WT scAAV6 vectors in CD34^+^ cells *in vitro*. We also document that the transgene expression from the B19p6 is significantly higher than that from the HS2-βp, and can be further increased following erythroid-differentiation. Expression from the B19p6 in the Y705+731F double-mutant scAAV6 vectors is also significantly higher than that from the HS2-βp in human CD34^+^ cells in a murine xenograft model *in vivo*. Transgene expression was detectable up to 12 weeks post-transplantation in primary recipients, and up to 6 additional weeks in secondary transplanted animals. These data suggest that the Y705+731F scAAV6-B19p6-βb-globin vectors might prove to be useful for the potential gene therapy of human hemoglobinopathies in general, and β-thalassemia and sickle cell disease in particular.

## Materials and Methods

### Cell Lines, Cells, and Cell Cultures

Human embryonic kidney 293 (HEK293) and erythroleukemia K562 cells were obtained from American Type Culture Collections (Manassas, VA) and maintained in Dulbecco’s-modified Eagle’s medium (DMEM; Lonza, Walkersville, MD), or Iscove’s-modified Dulbecco’s medium (IMDM; Irvine Scientific, Santa Ana, CA) supplemented with 10% fetal bovine serum (FBS; Sigma, St. Louis, MO), 100 µg/ml of penicillin and 100 U/ml of streptomycin (Invitrogen, Grand Island, NY). Human cord blood CD34^+^ cells and CD36^+^ cells were purchased from AllCells (AllCells Technologies, Emeryville, CA) and maintained in StemSpan™ Serum-Free Expansion Medium (SFEM) (StemCell Technologies, Vancouver, BC, Canada) with 10 ng/ml of recombinant human interleukin 6 (rhIL6) (Peprotech), 10 ng/ml of Interleukin 3 (rhIL3) (Peprotech, Rocky Hill, NJ) and 10 ng/ml of recombinant human stem cell factor (rhSCF) (R&D Systems, Minneapolis, MN).

### Plasmids

Plasmid pACGr2c6 and plasmid pscAAV2-CBAp-EGFP were a kind gifts from Drs. R. Jude Samulski and Xiao Xiao, University of North Carolina at Chapel Hill, Chapel Hill, NC. Plasmid pscAAV2-HS2-βp-EGFP plasmid was constructed by replacing the CBAp in plasmid pscAAV2-CBAp-EGFP with the HS2-βbp from plasmid pHPV37, generously provided by Dr. Philippe LeBoulch, Harvard Medical School, Boston, MA. Tyrosine double-mutant (DM) plasmid pAAV-Y705+731F was generated by using QuikChange® II Site-Directed Mutagenesis Kit (Stratagene, Santa Clara, CA) as described previously [15], [17]. pscAAV-CBAp-GLuc containing Gaussia luciferase (Gluc) reporter gene was used to generate plasmid pscAAV-B19p6-Gluc, in which the CBA promoter was replaced by the B19p6 promoter from plasmid pscAAV-B19p6-Fluc by digesting with Mlu I and Age I restriction enzymes. Plasmid pscAAV-HS2-βp-Gluc was constructed using standard cloning methods. Briefly, the HS2-βp insert with Mlu I and Age I sites was first cloned by polymerase chain reaction (PCR) from plasmid pscAAV-HS2-βp-globin using following primers-pair: βp-Age I-F: 5′-GCGACCGGTGGTGTCTGTTTGAGGTTGCTA-3′; and HS2-Mlu I-R: 5′-CGACGCGTTCAGATCGATCTCTCCCCAGCAT-3′. The PCR product was cloned as an insert in plasmid vector pscAAV-CBAp-GLuc following digestion with Mlu I and Age I restriction enzymes and ligation.

### Viral Vector Production

Viral vectors were packaged using a protocol described previously [15], [17]. Briefly, HEK 293 cells were co-transfected with three plasmids using Polyethelenimine (PEI, linear, MW 25000, Polyscinces, Inc., Warrington, PA), and medium was replaced 4 hrs post-transfection. Cells were harvested 72 hrs post-transfection, subjected to 3 rounds of freeze-thaw and digested with Benzonase (Invitrogen, Grand Island, NY). Vectors were purified by iodixanol (Sigma, St. Louis, MO) gradient ultra-centrifugation followed by ion exchange chromatography using HiTrap Q HP (GE Healthcare, Piscataway, NJ), washed with phosphate-buffered saline (PBS) and concentrated by centrifugation using centrifugal spin concentrators with 150K molecular-weight cutoff (MWCO). Titers were determined by quantitative DNA slot blot using ^32^P-labeled specific DNA probes as previously described [15].

### Induction of Erythroid Differentiation and Evaluation of Gluc Expression *in vitro*

Epo is commonly used for CD34^+^ cells erythroid introduction [29]. In the current study, CD34^+^ cells (15,000 cell/well, 3 well/group) were cultured in SFEM containing IL-3 (10 ng/ml), IL-6 (10 ng/ml), SCF (10 ng/ml) and with or without Epo (3 U/ml). CD34^+^ and CD36^+^ cells were cultured in SFEM with 10 ng/ml of rhIL6, rhIL3, and 10 ng/ml of rhSCF with or without 3 U/ml of recombinant human Epo for up to 15 days. Surface glycophorin A (GPA) expression were determined by flow cytometry using PE conjugated anti-GPA antibodies. K562 cells were seeded at a density of 1×10^5^ cells/ml in 6-well plates and cultured for 4 days in the presence or absence of 3 U/ml of Epo or 0.6 mM sodium butyrate or 400 µM hydroxyurea (HU). Benzidine staining was used to determine hemoglobin synthesis. Briefly, 100 µl of 0.2% benzidine (Sigma, St. Louis, MO) staining buffer prepared in 0.5 M glacial acetic acid was added to 100 µl cells; and then 5 µl of 30% hydrogen peroxide (H_2_O_2_) was added to the mixture; after incubation at RT for 10 minutes, the proportion of benzidine-positive cells was quantified using a hemocytometer under a light microscope.

For Epo-induction, equivalent numbers of K562 cells, CD34^+^, and CD36^+^ cells were cultured as described above, and at indicated time-points, were either mock-infected, or infected with scAAV6-Gluc vectors under the control of CBAp, HS2-βp, or B19p6 promoters, respectively, for 2 hrs. Gluc activity in the medium was examined at 18 hrs post-infection using commercially available BioLux® Gaussia Luciferase Flex Assay Kits (New England Biolabs, Inc, Ipswich, MA) with an injector-equipped luminometer (BMG Labtech, FLUOstar Optima, Cary, NC).

### Xenotransplantation

Equivalent numbers of human cord blood-derived CD34^+^ cells were cultured in four round-bottom 15×75 mm Falcon tubes (BD Biosciences) and either mock-infected, or infected with 2×10^4^ vgs/cell of WT-scAAV6-B19p6-Gluc, DM-scAAV6-HS2-βp-Gluc, or DM-scAAV6-B19p6-Gluc vectors for 2 hr at 37°C. Tubes were gently shaken every 15 min during infection. Cells were then washed and resuspended at a cell density of 5×10^6^/ml in DPBS prior to transplantation.

Five to 12-week-old NOD.Cg-*Prkdc* ^scid^ Il2rgtm1Wjl/SzJ (NSG) female recipient mice were used in the present study since female mice have been reported to be more efficient recipient than male mice for engraftment of human HSCs [30]. Mice were bred and kept in microisolator cages in the SPF facility at the University of Florida. Antibiotics were administrated by supplementing the drinking water with 0.2 mg/ml enrofloxacin (Bayer Healthcare, Shawnee Mission, KS) for 2 days before performing transplantation and 2 weeks post-transplantation to prevent infection. Mice were irradiated with a dose of 250 cGY from a Cesium-137 source at 4 hrs before injecting the mock- or AAV vector-transduced human cord blood-derived CD34^+^ cells. Approximately 1×10^6^ cells were injected into the lateral tail vein of each mouse. All animal experiments were approved by the University of Florida Institutional Animal Care and Use Committee. All procedures were carried out in accordance with the principles of the National Research Council’s Guide for the Care and Use of Laboratory Animals. All efforts were made to minimize suffering.

### Evaluation of Transgene Expression in Peripheral Blood

The method to evaluate Gluc activity was modified from the protocol described previously [31], [32]. Coelenterazine free base, the Gluc substrate, was purchased from Nanolight Technology, Pinetop, AZ. To prepare the stock solution, one drop of concentrated HCl was added to 10 ml of methanol to make acidified methanol. The corresponding amount of acidified methanol (2 ml) was then added to coelenterazine (10 mg) in an amber vial to make 5 mg/ml (∼12 mM) substrate solution. Stock solution was aliquoted and stored at −80°C. For *in vitro* blood Gluc activity assay, the stock solution was freshly diluted to 100 mM in PBS supplemented with 5 mM NaCl (pH 7.2). Mice were restrained with the tail exposed. The lateral tail vein was punctured using a 1 ml insulin needle; five to 20 µl of blood was collected using 20 µl tips. Samples were collected in anticoagulant tube in the presence of EDTA as an anticoagulant and placed on ice until all samples were collected. Blood samples were transferred to a 96-well plate, and the Gluc activity was measured using a plate luminometer (BMG Labtech, FLUOstar Optima, Cary, NC). Data were analyzed by plotting the relative light units (RLU) per second.

### *In vivo* Bioluminescence Imaging

Mice were weighed to calculate the volume of substrate according to the dose of 4 mg/kg of body weight and anesthetized. The calculated volume of the 5 mg/ml of stock substrate solution was mixed with 100 µl of PBS and injected via retro-orbital route [31]. *In vivo* bioluminescence images were acquired immediately over a period of 5 min using a Xenogen IVIS® Lumina II (Caliper Life Sciences) equipped with a cooled couple-charged device (CCD) camera (PerkinElmer Co., Alameda CA). Signal intensity was quantified using the camera control program, Living Image software version 4, and shown as photons/second/cm^2^/steridian (p/s/cm^2^/sr).

### Cell Sorting, Lineage Analyses, and Transgene Expression

Twelve-weeks post-transplantation of human CD34^+^ cells in primary recipient NSG mice, bone marrow cells were flushed from the bones of the hind limb with sterile PBS. Red blood cells were hemolyzed with ammonium chloride buffer. Cells were then labeled with fluorescein isothiocyanate (FITC) conjugated anti human CD45 and allophyocyanine (APC) conjugated anti mouse CD45 antibodies, and the percentage of human CD45-positive cells was calculated. For sorting of lineage specific cells, the bone marrow cells were labeled with FITC-conjugated anti human CD71 for erythroid, phycoerythrin (PE)-conjugated anti human CD19 for B cells, and APC-conjugated anti-human CD11b for monocytes and neutrophils. All antibodies were from BD Biosciences (San Jose, CA). Each lineage-specific cells were sorted using BD Aria TMIIu Fluorescence-Activated Cell Sorter (BD Biosciences). For determining Gluc activity in the sorted cell populations, ∼4×10^4^ cells from each lineage were suspended in 100 ml PBS. Five ml of the cell mixtures were used for the *in vitro* Gluc activity assay as described above.

### Secondary Transplantation

Twelve-weeks post-primary transplantation, the whole bone marrow cells from a mouse transplanted with human CD34^+^ cells transduced with DM-scAAV6-B19p6-Gluc vectors were isolated as described above. Approximately 2×10^6^ bone marrow cells were transplanted into NSG mice (n = 4) via retro-orbital injection following irradiation with 250 cGy. Mice were maintained on 0.2 mg/ml enrofloxacin in drinking water (Bayer Healthcare, KS). Six-weeks post secondary transplantation, mice were subjected to whole-body bioluminescence imaging *in vivo* as described above.

## Results

### Transduction Efficiency of Single- and Double-tyrosine Mutant scAAV6 Serotype Vectors in Human Hematopoietic Cells *in vitro*

**Figure 1:**
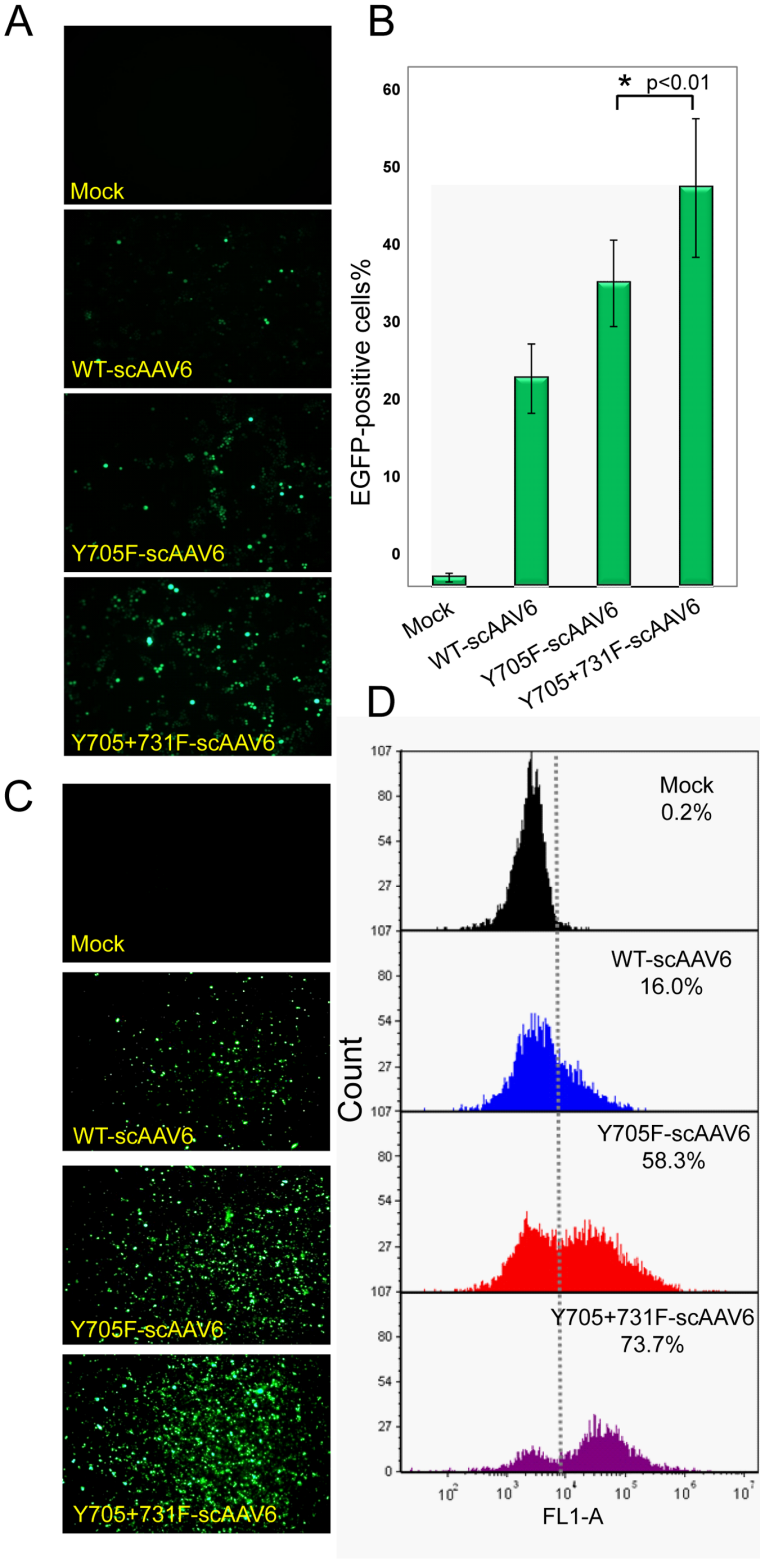
Transduction efficiency of WT and tyrosine-mutant scAAV6 vectors in human hematopoietic cells. Approximately 5×10^3^ K562 cells were either mock-infected, or infected with 5×10^3^ vgs/cell of WT or various tyrosine-mutant scAAV6-CBAp-EGFP vectors, and transgene expression was determined 48 hrs post-infection using a Zeiss fluorescence microscope (Panel A), and Accuri C6 flow cytometer (Panel B) (Original magnification, x100) Approximately 1×10^4^ primary human CD34^+^ cells were either mock-infected, or infected with 2×10^4^ vgs/cell of WT or various tyrosine-mutant scAAV6-CBAp-EGFP vectors under identical conditions, and transgene expression was determined 72 hrs post-infection by fluorescence microscopy (Panel C) (Original magnification, x200), and quantified by fluorescence-activated cell sorting (FACS) using a BD FACS Aria Flow Cytometer followed by processing with software FCS Express 4 (Panel D). *Y705+731F-scAAV6 vs. WT-scAAV6 vectors, *p*<0.01.

We recently identified AAV6 as the most efficient serotype in transducing primary human HSCs, and that site-directed mutagenesis of specific surface-exposed tyrosine residues (Y705 and Y731) further increased the transduction efficiency of these vectors. Since we have previously reported that the transduction efficiency of AAV2 and AAV3 serotype vectors could be further improved by combining the single tyrosine-mutations, we wished to evaluate the transduction efficiency of the Y705F+Y731F double-mutant (DM) scAAV6 vectors. Both human erythroleukemia K562 cells, and primary human CD34^+^ cells were either mock-infected, or infected with WT, or single (Y705F)-, or DM (Y705+731F) scAAV6 vectors. K562 cells were infected with 5×10^3^ vgs/cell, and human CD34^+^ cells were infected with 2×10^4^ vgs/cell. Transgene expression was determined by fluorescence microscopy and quantified by flow cytometry. These results are shown in Figure 1. As can be seen, the transduction efficiency of DM scAAV-CBAp-enhanced green fluorescent protein (EGFP) vectors was significantly higher than that of either WT or single-mutant scAAV6 vectors, both in K562 cells (Panels A and B) and in CD34^+^ cells (Panels C and D). The percentage of EGFP-positive cells increased from 24.0±4.0% to 46.0±6.1% in K562 cells, and from 16.0±2.0% to 73.7%±5.1% in CD34^+^ cells.

### Transcriptional Potential of CBAp, HS2-βbp, and B19p6 Promoters

**Figure 2:**
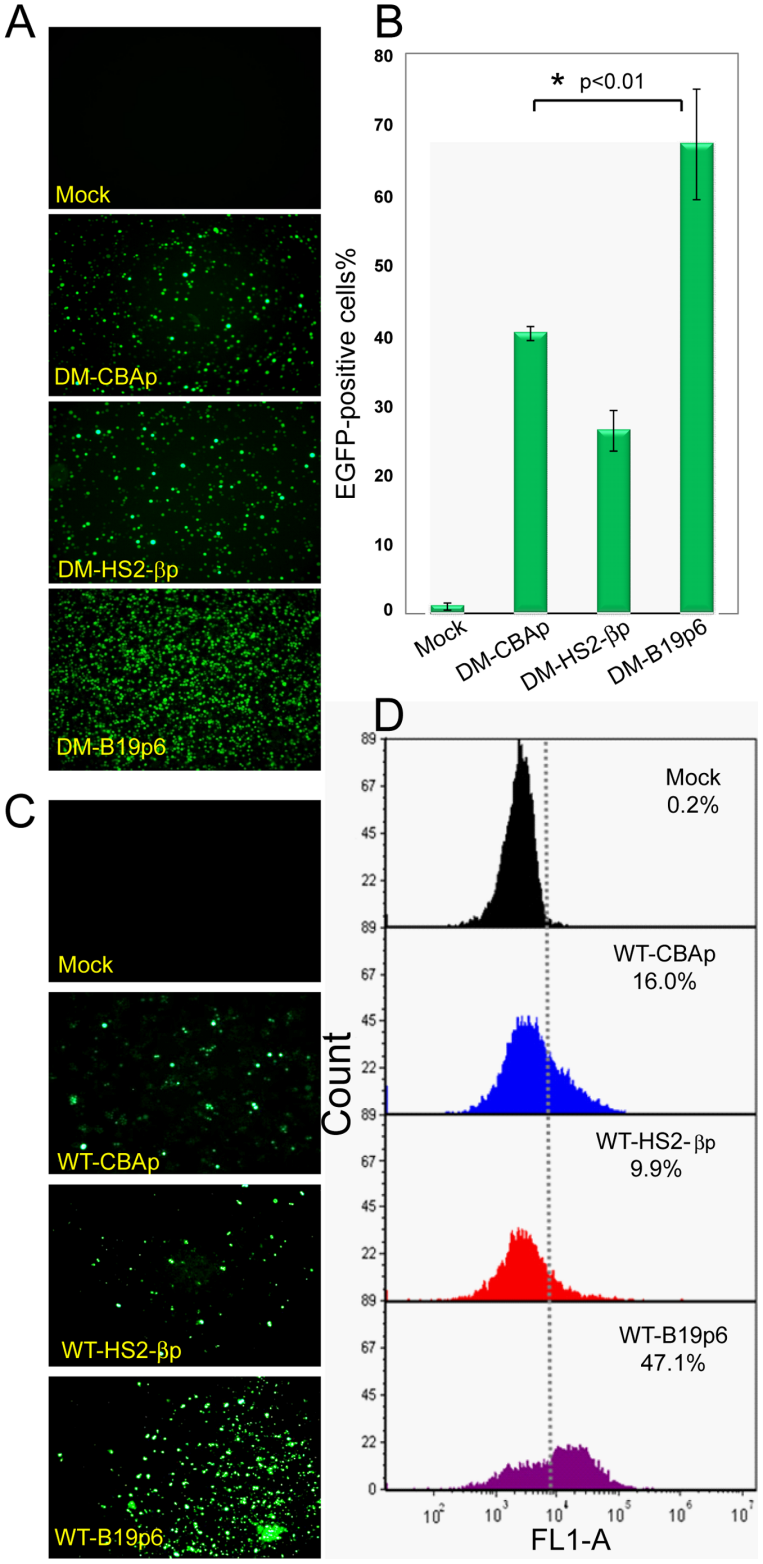
Transcriptional potential of CBAp, HS2-βp, and B19p6 promoters in human hematopoietic cells. Approximately 1×10^4^ cells were either infected with DM scAAV6 vectors (K562 cells) or WT scAAV6 vectors (human CD34^+^ cells) expressing the EGFP gene under the control of the three different promoters at 5×10^3^ vgs/cell (K562 cells) or 2×10^4^ vgs/cell (human CD34^+^ cells), respectively. Transgene expression in K562 cells (Panels A and B) and human CD34^+^ cells (Panels C and D) was determined 72 hrs post-infection by fluorescence microscopy and quantitated by the flow cytometry as described above. The original image magnifications were 100× (Panel A) and 200× (Panel B). *DM-scB19p6-EGFP vs. DM-scAAV6-HS2-βp-EGFP vectors, *p*<0.01.

With the ultimate objective of developing recombinant AAVs vectors for the potential gene therapy of human hemoglobinopathies, we next evaluated the transcriptional potential of the following two erythroid cell-specific promoters: HS2-βp, and the B19p6. The ubiquitous CBAp was used as an appropriate control. scAAV6 vectors containing the EGFP gene under the control of the three promoters were used to infect primary human CD34^+^ cells under identical conditions, and transgene expression was evaluated 72 hrs post-infection. These results are shown in Figure 2. In K562 cells (Panels A and B), the EGFP expression level from DM-scAAV6-B19p6-EGFP vectors was ∼67.0±7.9%, which is significantly higher that from DM-scAAV6-CBAp-EGFP vectors (∼40.0±1.0%), and from DM-scAAV6-HS2-βbp-EGFP vectors (∼26.1±2.9%). Similarly, in human CD34^+^ cells (Panels C and D), whereas little transgene expression occurred in mock-infected cells, ∼16% of cells transduced with scAAV6-CBAp-EGFP vectors were EGFP-positive. Transgene expression from scAAV6-HS2-βbp-EGFP occurred in ∼10% of cells, whereas ∼47% of cells transduced with scAAV6-B19p6-EGFP vectors were EGFP-positive.

### Transgene Expression from the CBAp, HS2-βbp, and B19p6 Promoters following Erythroid Differentiation *in vitro*

**Figure 3:**
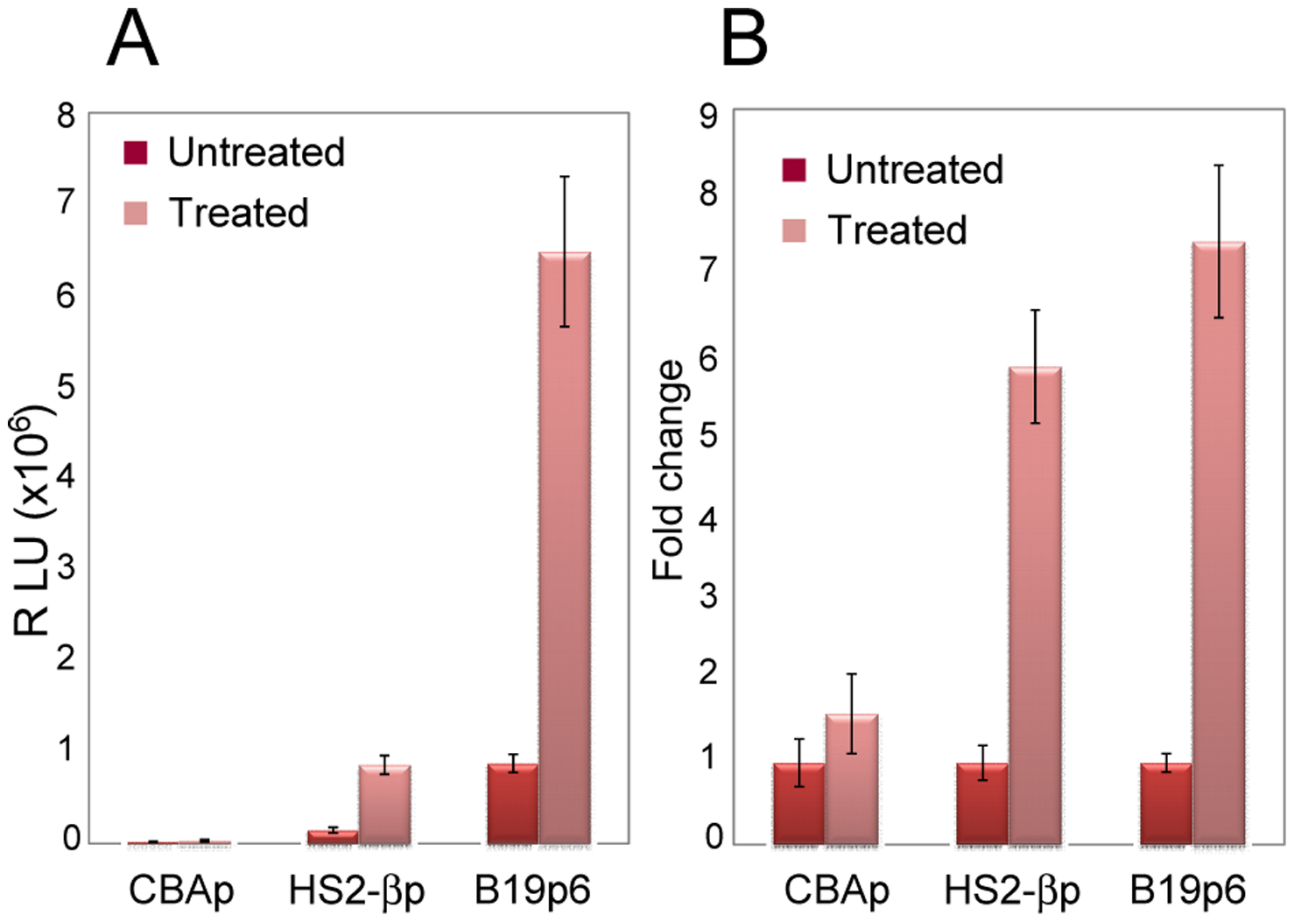
Transcriptional potential of CBAp, HS2-βp and B19p6 promoters in human erythroleukemia cells following erythroid differentiation. Equivalent numbers of mock-treated, or Epo-induced erythroid-differentiated K562 cells were infected with 5×10^3^ vgs/cell of scAAV6-Gluc vectors, and transgene expression was determined 18 hrs post-infection (A). Fold changes in transgene expression from the three promoters were calculated from untreated vs. Epo-treated groups (B).

Since HS2-βp and B19p6 are erythroid cell-specific promoters, we wished to examine whether transgene expression from these promoters could be further increased following erythroid differentiation of K562 cells. K562 cells were cultured for 4 days in the presence or absence of the 3 U/ml of erythropoietin (Epo), and equivalent numbers of cells were either mock-infected, or infected with 5×10^3^ vgs/cell of Y705+731F DM-scAAV6 vectors expressing the Gluc reporter gene under the control of the CBAp, HS2-βp, or the B19p6 promoters as described in Materials and Methods. Gluc activity was determined 18 hrs post-infection. As can be seen in Figure 3, transgene expression from B19p6 promoter in untreated K562 cells was significantly higher than that from CBAp and HS2-βp promoters (Panel A). The extent of transgene expression from both HS2-βp and B19p6 increased up to 7-fold following Epo-induced differentiation, whereas no significant change was observed from the CBAp, with or without Epo-induced differentiation (Panel B). Similar results were observed with butyrate- or HU-induced erythroid differentiation of K562 cells (data not shown). However, since HU has been shown to increase the transduction efficiency of AAV vectors [33], and butyrate has been reported to be related to activation of p38 MAP kinase [34], which also affects AAV transduction [35], the observed increase in transgene expression may not solely be credited to erythroid differentiation.

**Figure 4:**
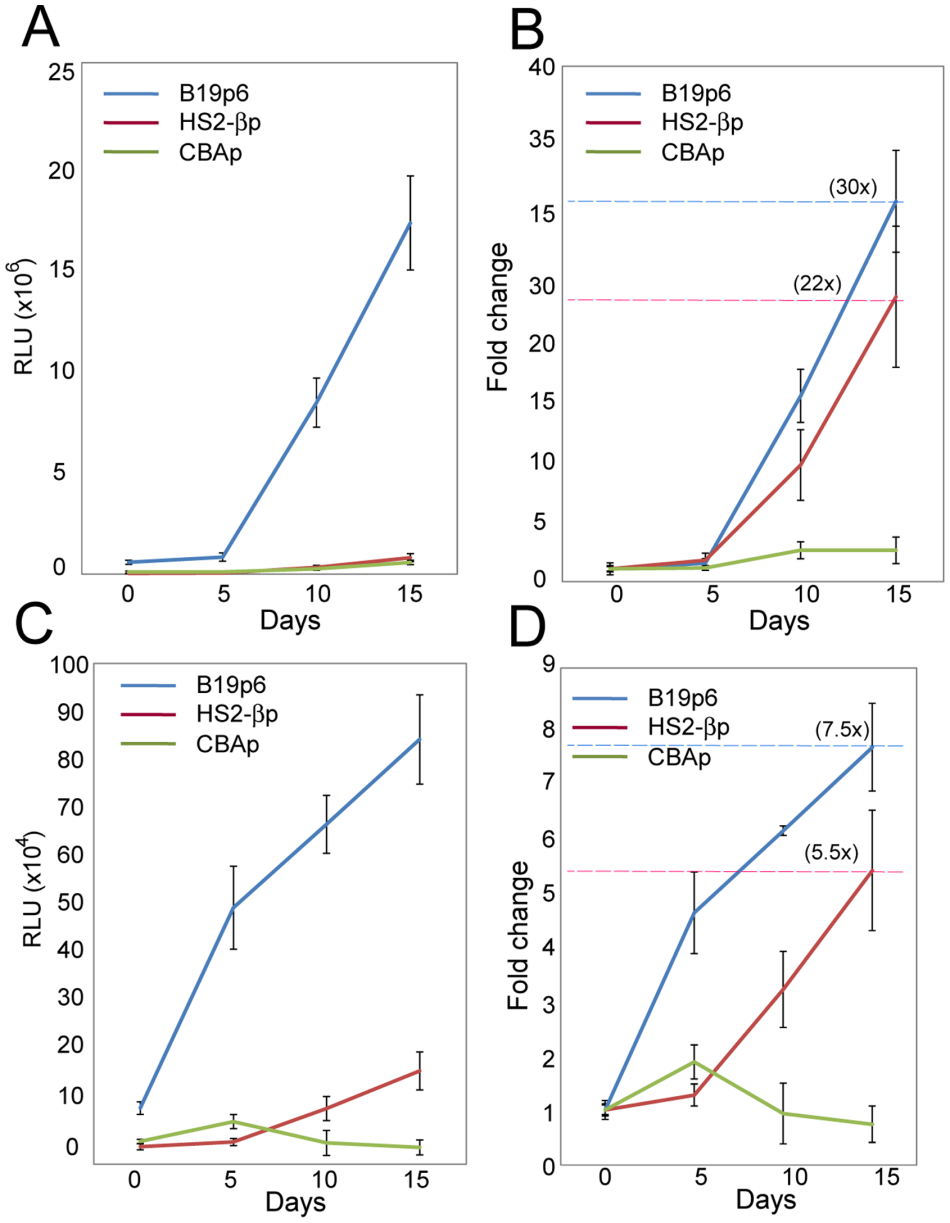
Transcriptional potential of CBAp, HS2-βp and B19p6 promoters in primary human CD34^+^ and CD36^+^ human cells following erythroid differentiation. Approximately 1.5×10^4^ CD34^+^ cells, and ∼2×10^4^ primary human CD36^+^ erythroid progenitor cells were cultured with or without Epo (3 U/ml) for various indicated times, and infected with 1×10^4^ vgs/cell scAAV6-Gluc vectors under identical conditions. Transgene expression levels were determined at 18 hrs post-infection at each time-point. Gluc activity at various time-points was normalized to the group without Epo-induction, and the normalized absolute values are shown as average ± standard deviation from triplicates for CD34^+^ cells (Panel A), and for CD36^+^ cells (Panel C). Fold changes in transgene expression following erythroid-differentiation were calculated by dividing the normalized Gluc activities by the initial activity on Day 0 (Panels B and D).

Epo is also commonly used to induce erythroid differentiation in CD34^+^ cells [29]. In the current study, CD34^+^ cells were cultured for various indicated days in the presence or absence of Epo, and equivalent numbers of cells were either mock-infected, or infected with 1×10^4^ vgs/cell of Y705+731F DM-scAAV6-Gluc vectors. Gluc activity was determined 18 hrs post-infection at each time-point. These results are shown in Figure 4 (Panels A and B). As is evident, transgene expression from both HS2-βp and B19p6 kept increasing until the end of the experiments, whereas expression from the CBAp remained unaffected, the extent of transgene expression from the HS2-βp and the B19p6 promoters increased up to 22-fold and 30-fold, respectively, 15 days post-erythroid differentiation. Similar results were obtained with primary human CD36^+^ erythroid progenitor cells (Figure 4, Panels C and D). The extent of transgene expression from the HS2-βp and the B19p6 promoters increased up to 5.5-fold and 7.5-fold, respectively, 12 days post-erythroid differentiation.

### Transgene Expression from the HS2-βp and B19p6 Promoters in a Xenograft Murine Model *in vivo*

Transgene expression from the HS2-βp and the B19p6 promoter was also evaluated in an immuno-deficient xenograft mouse model *in vivo*. Female non-obese diabetic [36] severe combined immune-deficient (SCID), gamma (NSG) mice have been reported to be a good xenotransplantation model for assaying human cell engraftment [37]. In our present study, ∼1×10^6^ primary human CD34^+^ cells were either mock-infected, or infected by WT or Y705+731F DM-scAAV6 vectors expressing Gluc under the control of HS2-βp or the B19p6 promoters, respectively.

**Figure 5:**
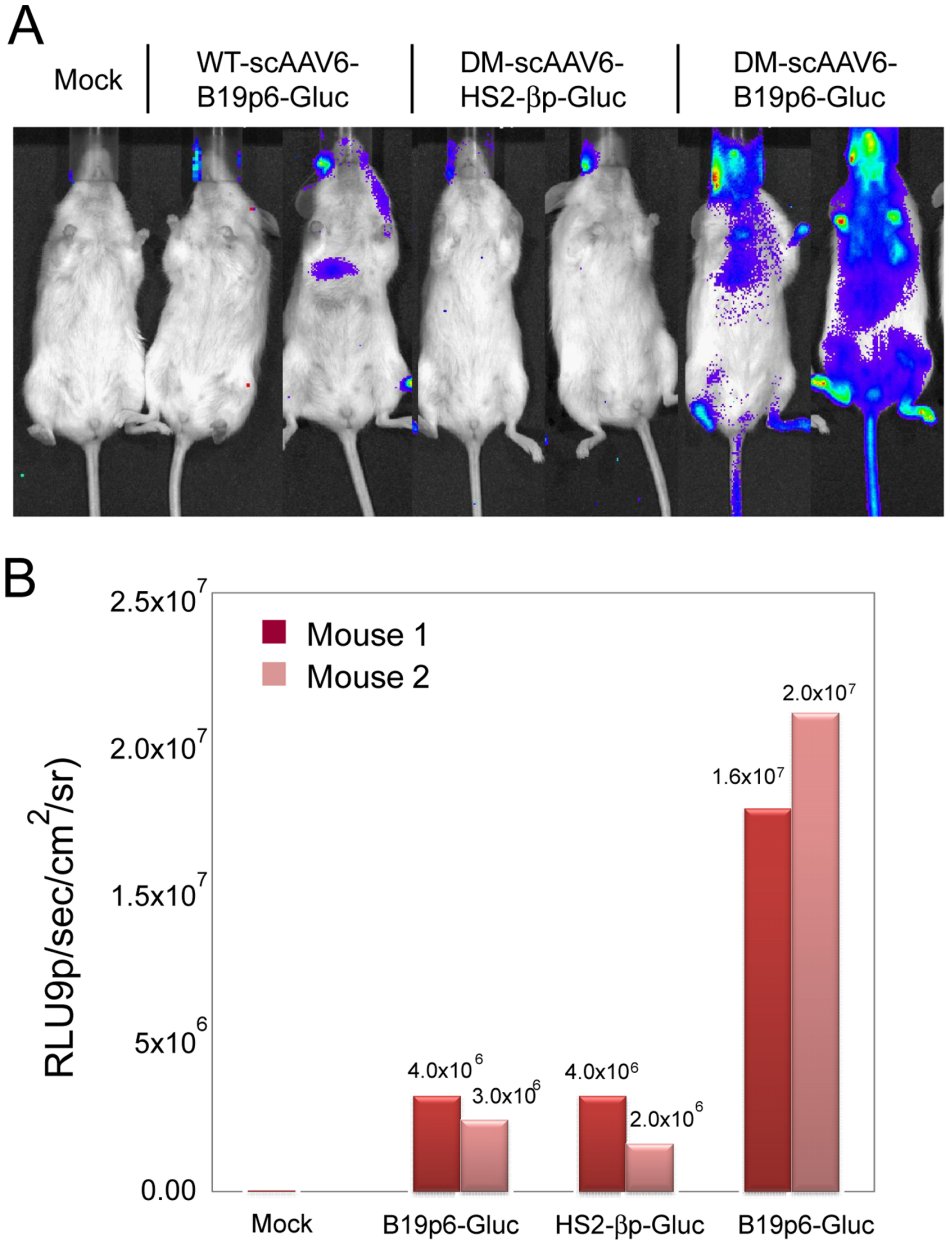
Bioluminescence imaging of mice transplanted with human CD34^+^ cell *in vivo*. **NSG**mice transplanted with mock-infected, or various indicated scAAV6 vector-infected primary human CD34^+^ cells was acquired by a Xenogen IVIS® Imaging System 6-weeks post-transplantation. Images of representative animals from each group are shown (Panel A). The luminescence signal intensity was quantified as photons/second/cm^2^/steridian (p/s/cm^2^/sr) using the Living Image® software (Panel B).

Whole-body bioluminescence images (Figure 5, Panel A), acquired as detailed in Materials and Methods at 6 weeks post-implantation, corroborated that whereas no transgene expression occurred in mice transplanted with mock-infected human CD34^+^ cells, expression from the B19p6 promoter in Y705+731F DM-scAAV6 vectors was up to 5-fold higher than that from the B19p6 promoter in WT scAAV6 vectors, or from the HS2-βbp promoter in Y705+731F DM-scAAV6 vectors (Figure 5, Panel B).

**Figure 6:**
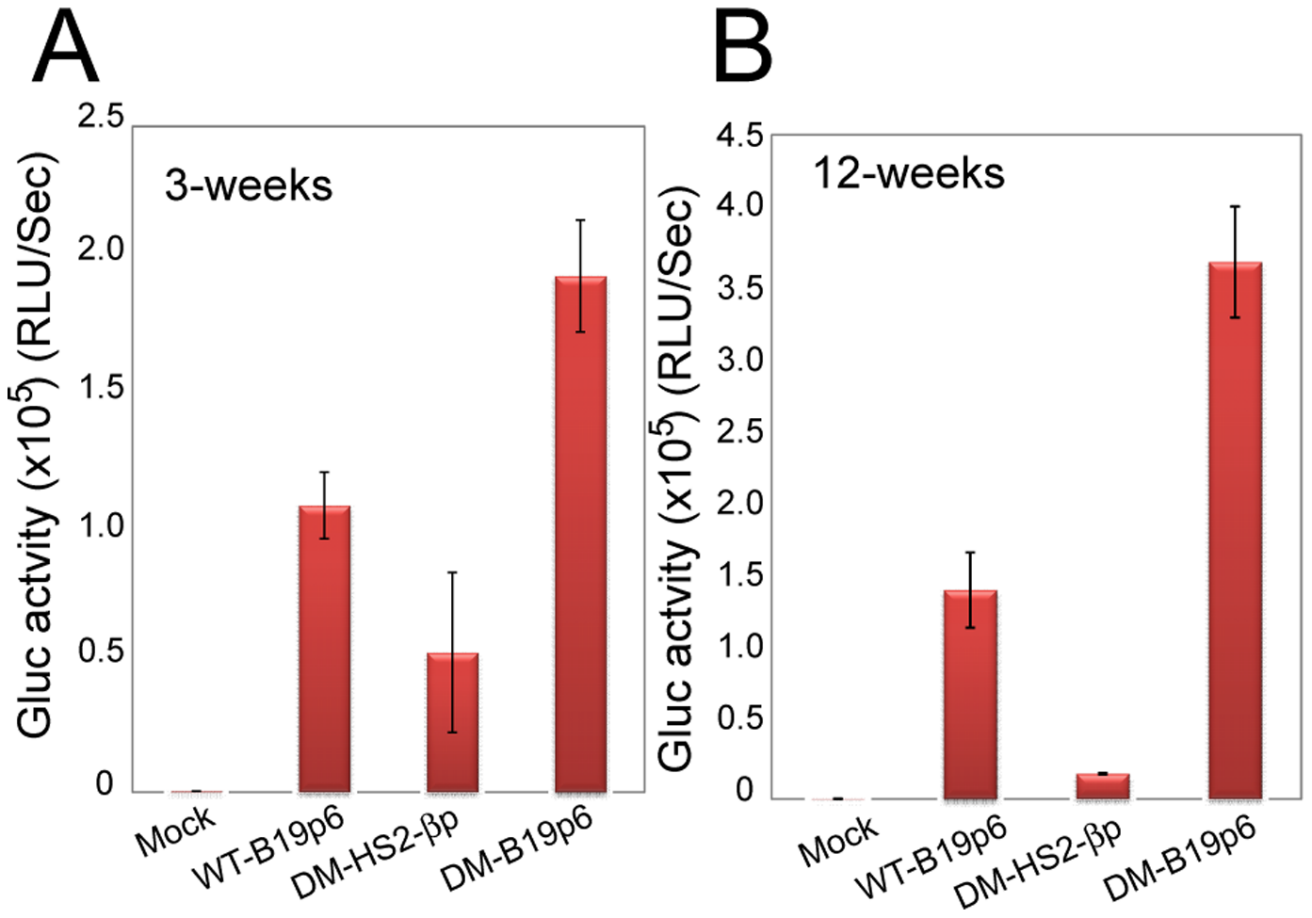
Relative levels of transgene expression from HS2-βp and B19p6 promoters in primary human CD34^+^ cells following xenotransplantation in NSG mice. Approximately 1×10^6^ primary human CD34^+^ cells were either mock-infected, or infected with 2×10^4^ vgs/cell of WT-scAAV6-B19p6-Gluc, Y705+731F DM-scAAV6-HS2-βbp-Gluc, or Y705+731F DM-scAAV6-B19p6-Gluc vectors under identical conditions, and engrafted into NSG mice as described under Materials and Methods. Gluc activity was measured 3 weeks (Panel A) and 12-weeks (Panel B) post-engraftment in peripheral blood using a luminometer. Total relative light units (RLU) per second were calculated, and results are presented as mean ± s.d., with P<0.001 as calculated by student’s t-test.

Since Gluc is secreted and it has very high tissue absorption, human CD34^+^ cell engraftment in NSG mice and transgene expression levels were monitored *in vivo* both by Gluc activity in peripheral blood (3 weeks and 12 weeks post-transplantation). As can be seen in Figure 6A, Gluc expression from the B19p6 promoter in the WT scAAV6 vectors was >2-fold higher than that from the HS2-βp promoter in the Y705+731F double-mutant scAAV6 vectors, and expression from the B19p6 promoter in the Y705+731F double-mutant scAAV6 vectors was ∼4-fold higher than that from the HS2-βbp promoter in Y705+731F double-mutant scAAV6 vectors in peripheral blood in NSG mice 3 weeks post-transplantation (Panel A). The extent of transgene expression was further increased from the B19p6 promoter 12 weeks post-transplantation (Panel B).

**Figure 7:**
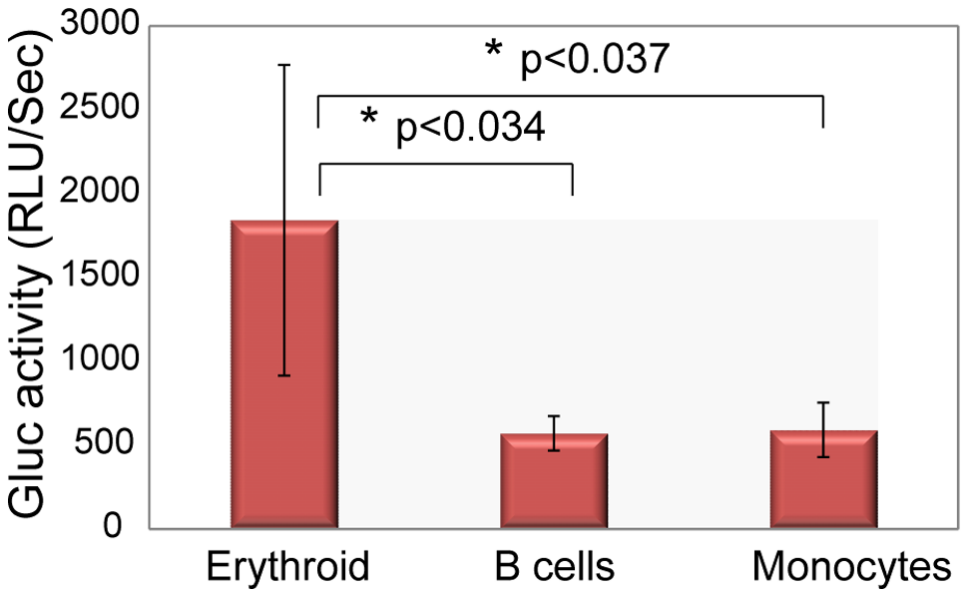
Transgene expression in various human hematopoietic lineages 12-weeks post-transplantation of human CD34^+^ cells in primary recipient NSG mice. Bone marrow cells were harvested and human lineage-specific cells were sorted and Gluc activity in the sorted cell populations, was determined as described above. *p<0.034 (erythroid vs. B cells) and *p<0.037 (erythroid vs. monocytes).

In order to evaluate whether the observed transgene expression from the B19p6 promoter was restricted to human erythroid progenitor cells, whole bone marrow cells were harvested from primary recipient mice 12 weeks post-transplantation. Anti-human antibodies were used to sort for erythroid, B cells, and monocytes using lineage-specific antibodies as described under Materials and Methods. Gluc activity in the sorted cell populations was determined as described above. These results, shown in Figure 7, suggest that transgene expression from the B19p6 promoter is largely restricted to human erythroid progenitor cells.

**Figure 8:**
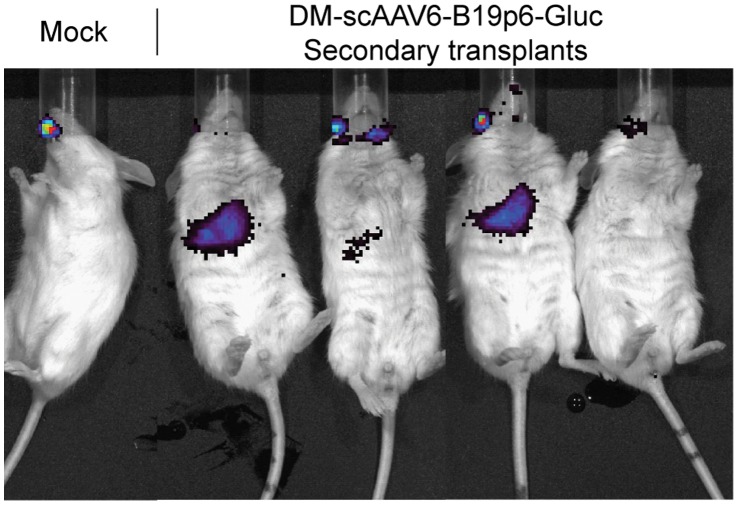
Bioluminescence imaging of mice following secondary transplantation. Whole bone marrow cells from NSG mice transplanted with mock-infected, or DM scAAV6-B19p6-Gluc vector-infected primary human CD34^+^ cells were harvested 12-weeks post-primary transplantation, and transplanted into secondary recipient mice. Six-weeks post secondary transplantation, mice were subjected to whole-body bioluminescence imaging *in vivo* as described above.

To further evaluate whether long-term repopulating human stem cells were transduced, whole bone marrow cells from a mouse transplanted with human CD34^+^ cells transduced with DM-scAAV6-B19p6-Gluc vectors were isolated 12-weeks post-transplantation, and transplanted into secondary recipient mice (n = 4). Whole-body bioluminescence imaging *in vivo* was performed 6-weeks post-secondary transplantation as described above. As can be seen in Figure 8, transgene expression was observed in each animal, albeit at low levels, due to <1% engraftment of human cells (data not shown). These results, nonetheless, document that DM scAAV6 vectors are capable of transducing long-term repopulating human stem cells.

## Discussion

An ideal gene therapy vector for the potential gene therapy of b-thalassemia and sickle cell disease would be one with which high-efficiency transduction of primitive human HSCs could be achieved, and following erythroid differentiation, robust levels of expression of the transduced human b-globin gene could be obtained. The development of lentiviral vectors by a number of investigators has indeed achieved these objectives [38]–[45], but their long-term safety still remains an open question [1]. We and others have described the development of the first generation of recombinant AAV2 vectors for the potential gene therapy of b-thalassemia and sickle cell disease [4]–[8], but in retrospect, it has become clear that the use of the WT AAV2 capsid, and the single-stranded nature of the vector genome, were major obstacles to achieving therapeutic levels of the human b-globin gene [6], [8], [46]–[48]. In addition, the use of murine models of these diseases was not predictive of the potential efficacy of a number of alternative serotypes of AAV vectors [27], [28].

Based on more recent studies by us and others [12]–[14], in which AAV6 was identified to be the most efficient serotype for transducing human HSCs, and our observation that B19p6 is more robust than HS2-βp for mediating erythroid lineage-restricted expression [27], we reasoned that combining these features might lead to the development of an ideal vector for the potential gene therapy of β-thalassemia and sickle cell disease, especially since the safety and efficacy of AAV vectors have now been established unequivocally in a number of Phase I/II clinical trials in humans [49]–[53]. Indeed, the Y705+731F double-mutant scAAV6 vectors containing the B19p6, described here, were determined to be the most efficient in transducing primary human CD34^+^ cells, and mediating erythroid lineage-restricted transgene expression, both *in vitro* and *in vivo*. It is also possible that the transduction efficiency of these vectors can be augmented further, based on our recent observations that site-directed mutagenesis of specific surface-exposed serine and threonine residues improves the transduction efficiency of AAV2 serotype vectors [35], and most of these residues are highly conserved in all AAV serotypes.

The basic underlying molecular mechanism of increased transduction efficiency of the Y705+731F double-mutant scAAV6 vectors in human CD34^+^ cells is not readily apparent. Based on our recent studies with tyrosine-mutant AAV2 and AAV3 serotype vectors [15], [17], we favor the hypothesis that improved intracellular trafficking and/or nuclear transport lead to the observed effect. However, the alternative hypothesis that a more efficient cellular receptor-mediated viral vector entry also play a role, cannot be ruled out since the extent of transgene expression from the B19p6 promoter in human CD36^+^ erythroid progenitor cells was ∼20-fold lower than the more primitive CD34^+^ HSCs (compare Figures 4 A, B and Figure 4 C, D). Thus, additional studies are warranted to address these issues, as well as to identify the authentic receptor for AAV6 for human HSCs, since in a recent report, EGFR was recently identified to be the cellular receptor for AAV6 [54], and Denard *et al*. [55] reported that galectin 3-binding protein in human sera agglutinates AAV6 vectors, which resulted in decreased transduction efficiency of these vectors. In our studies, pre-treatment of CD34^+^ cells with EGF had no effect on the transduction efficacy of AAV6 vectors, and K562 cells, which are known to lack expression of EGFR [56], could be efficiently transduced by AAV6 vectors, which was inhibited by FBS (data not shown).

Although erythroid lineage-restricted transgene expression from the B19p6 promoter in primary human HSCs *in vitro* has previously been reported [26], those studies were carried out with the first generation single-stranded AAV2 serotype vectors, which were clearly sub-optimal. We subsequently utilized scAAV1 and scAAV7 serotype vectors, and corroborated the erythroid cell-specificity of the B19p6 promoter *in vivo* [27], [28], those studies were carried out in murine HSCs, which were clearly not predictive for human HSCs. In the present studies, we documented sustained transgene expression in human HSCs, both in primary as well as in secondary transplant recipient mice. However, because of less than 1% engraftment of human cells in secondary transplant recipients, we were unable to document stable integration of the AAV proviral genomes. In this context, it is important to emphasize that the general conclusion that AAV genomes do not integrate, has largely been derived from previously published studies, all of which were carried out with post-mitotic cells and tissues, such as liver, muscle, brain, and retina, in which the AAV genomes remain episomal, although integration in liver has been reported by several investigators [57]–[60]. In our previously published studies with primary murine HSCs, stable integration of the AAV proviral genomes has been documented in both primary as well as secondary transplant recipient mice [7], [27], [28], [61], [62], and in a more recently published collaborative study, we have also documented long-term transduction and multi-lineage engraftment of human HSCs in a mouse xenograft model [63]. Thus, our working hypothesis has been that unlike in post-mitotic cells, AAV vectors do integrate in HSCs. The fact that in a recent report by Weltner *et al*. [64], all 4 reprogramming genes were shown to be integrated following AAV vector-mediated generation of induced pluripotent stem (iPS) cells, provides further support to our hypothesis.

The development of the optimized scAAV6-B19p6 vectors described here, with which high-efficiency transduction of human HSCs, and erythroid lineage-restricted expression can be achieved *in vivo*, and the possibility that the transduction efficiency of these vectors can be further augmented by introducing additional mutations in surface-exposed specific serine and threonine residues, similar to those described for AAV2 [35], [65], bodes well for the eventual use of these vectors in the potential gene therapy of human hemoglobinopathies in general, and b-thalassemia and sickle cell disease in particular.
